# Supplementary figures and images for: Sleep deprivation detected by voice analysis
Source: PLoS Comput Biol. 2024 Feb 5;20(2):e1011849. doi: 10.1371/journal.pcbi.1011849 (PMC10890756; doi:10.1371/journal.pcbi.1011849)

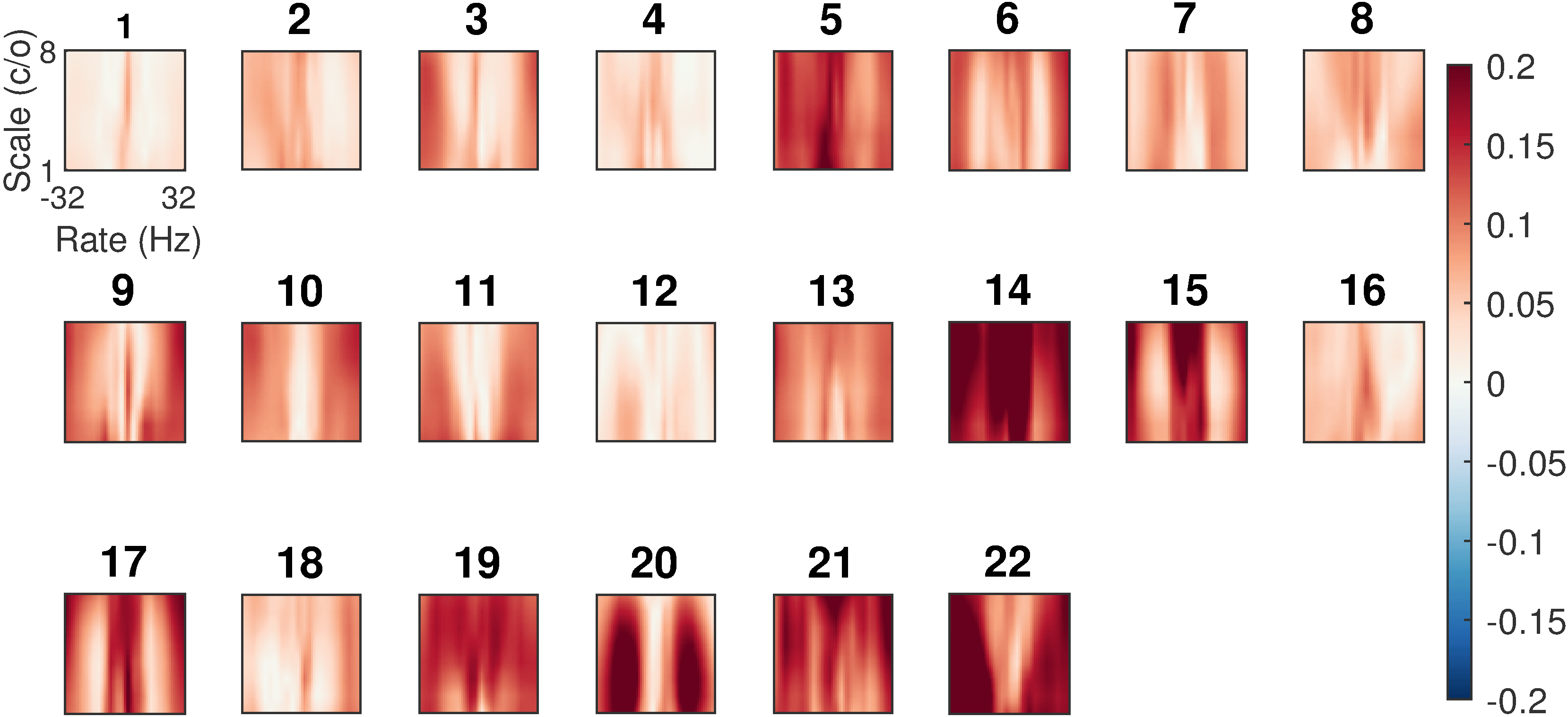

Supplement: S1 Fig — Units: Percent. (TIF) [file pcbi.1011849.s001.tif]

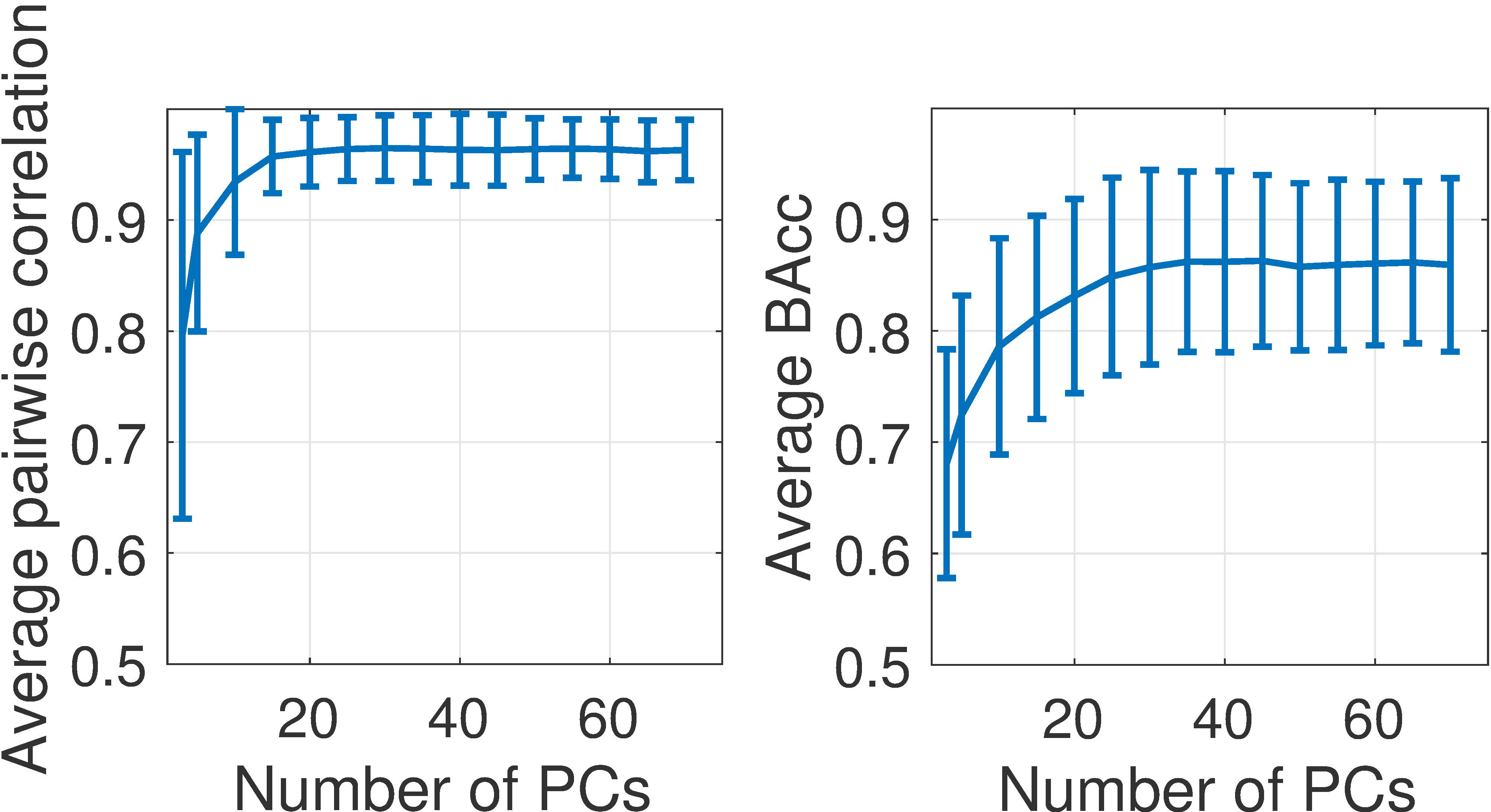

Supplement: S2 Fig — (Right) Average pairwise correlations between the interpretation masks for different number of Principal Components averaged across the 22 subjects. In order to evaluate for how many PCs the interpretations are stabilized, pairwise correlations between each masks, 1 one for each PC, has been done for each and averaged across the whole 22 subjects. We observe that the interpretation is stabilized around 20 PCs which. (Left) Error bars represent standard deviations. (TIF) [file pcbi.1011849.s002.tif]

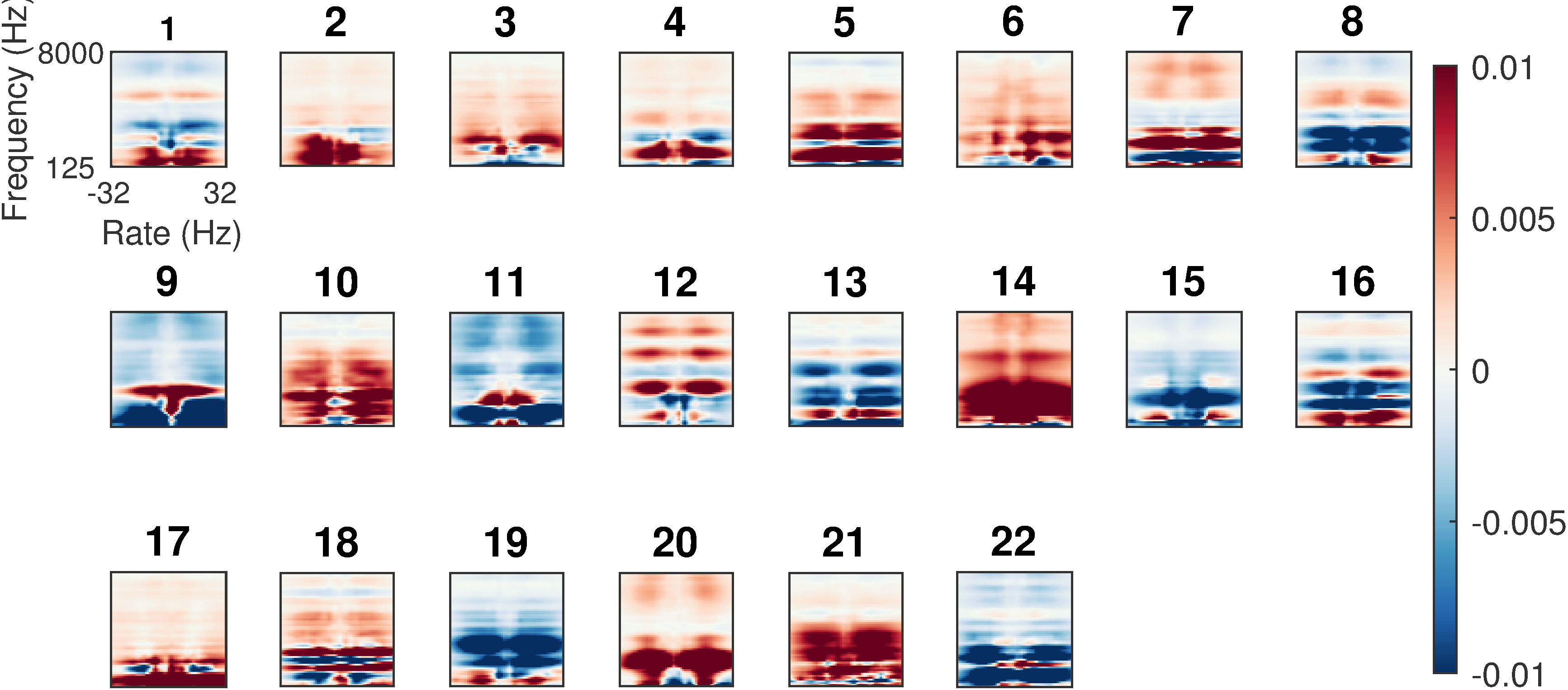

Supplement: S3 Fig — Red indicates the areas of the frequency rate projections that are used by classifier to predict a sleep deprived excerpt and conversely, blue indicates frequency-rate areas that characterize a non-sleep deprived voice. (TIF) [file pcbi.1011849.s003.tif]

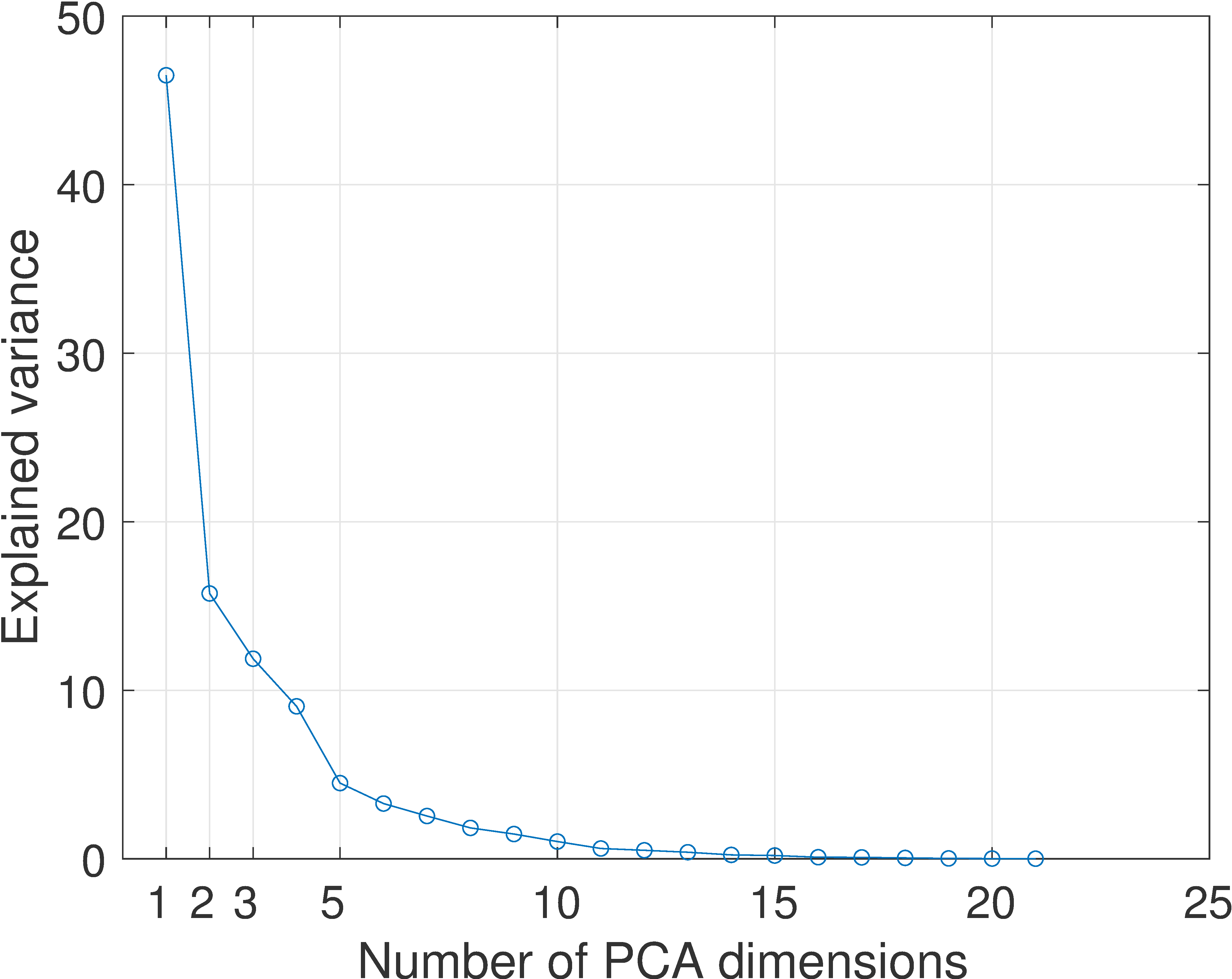

Supplement: S4 Fig — Units: percent. (TIF) [file pcbi.1011849.s004.tif]
