# Supplementary material for: Sleep deprivation detected by voice analysis
Source: PLoS Comput Biol. 2024 Feb 5;20(2):e1011849. doi: 10.1371/journal.pcbi.1011849 (PMC10890756; doi:10.1371/journal.pcbi.1011849)
Supplement: Attachment — Submitted filename: response_to_reviewers.pdf [file pcbi.1011849.s005.pdf]

Rebuttal letter

PCOMPBIOL-D-23-00407

Reviewer #1

This article discusses machine learning as a useful tool for objectively evaluating sleep deprivation. Overall, the manuscript is extremely well written, with substantial motivation toward the use of voice recordings as a simple, non-invasive, non-costly method to assess sleep deprivation. I have a series of minor questions and grammatical changes to improve the significance and readability of the work:

We would like to thank the reviewer for their appreciation of the manuscript and for the very helpful comments they provided. We have addressed each comment in full, as detailed below. We believe this has contributed to improve the manuscript in its form and content.

1. Conclusions of an inflammatory response in the throat and nose is a huge jump from altered timbre. For instance, these results could also be the effect of reduced or increased amplitude of vocal tract movement, such as in lazy or mumbled speech vs. clear speech, or perhaps due to differences in hydration pre/post sleep deprivation. To be clear, inflammatory response is one possibility, but it is also possible that these results are due to effects from the third-variable problem.

We agree with the alternative possibilities raised in your comment. These possibilities are now described in the Discussion. The abstract has been fully reworked to diminish the emphasis on our speculative hypotheses. Moreover, also to address a similar comment made by Reviewer 2, we now explicitly state in the Discussion that any physiological interpretation of the timbre changes observed after deprivation must remain tentative, because we did not collect the specific objective measures required to disentangle the different possibilities. Nevertheless, what the study allows is to formulate new hypotheses, which can be put to the test in future targeted investigations.

2. Could a lack of significant correlation between subjective sleepiness reports and timbre cues be the result of the third-variable problem (e.g., hydration, food intake) rather than (or in addition to) the hypothesized implicit inflammatory effect?

This is a distinct possibility, thank you for pointing it out. As we did not control for such variables, the possibility can only be acknowledged in the Discussion.

3. Although I agree with the reasoning for choosing an all-female experimental group, I am curious: Is there reason to suggest that these results are generalizable across sex (why or why not)?

As you point out, the motivation for choosing an all-female group was a trade-off between various constraints. As a result, the generalization of the present findings to a male population is unclear and would best be tested experimentally. Indeed, there are mixed reports of differences in the way sleep deprivation affects males or females, in particular relative to inflammation (Dolsen et al., 2019). We now mention this issue in the Discussion, together with a word of caution about generalizability.

4. Is there any correlation with results and participant age, typical amount of sleep per night, or usual bedtime?

All participants included in the study reported regular nighttime sleep between 7 and 8 hours on sleep logs, intermediate chronotype, and no sleep disorders based on a clinical interview. They also had to follow regular schedules the week before the survey. Thus, the amount of sleep and usual bedtime was quite homogeneous in the group, at the time of the survey. This limits the possibility to test, in our sample, for the correlations suggested. However, we hope that the practicality of the method we outline will enable future larger-scale investigations to address exactly such kinds of highly relevant questions in the future.

5. Why might [explain] the relatively poor accuracies resulted in 2/22 participants?

Following your question, we have looked for possible explanations of the two outliers for which poor sleep deprivation detection accuracy was observed. For one of them, sleepiness self-reports were on average lower after deprivation than before, a paradoxical pattern. This may be why the classifier failed to detect features of sleepiness in their voice. For the other one, self-reported sleepiness did increase after deprivation, so it could be that sleepiness did not impact their voice, or that, for whatever reason, the classifier failed to pick up the relevant features. Noticeably, the new classifier (new Fig 3) based on standard speech features also performed poorly with these two participants. We now mention these observations in the revised manuscript, directly in the Results presentation. Furthermore, the Discussion points out that interpreting negative findings is a general (and important) shortcoming of any objective measure of sleep deprivation.

Grammatical
Recommendations:
Line 60: increases (or can increase)
Line 150: as "of" yet
Line 294: consists of
Line 380: I recommend changing "vocal cords" to "vocal folds"

Done, thank you.

Reviewer #2

This manuscript describes an experiment by which speech recordings from 22 healthy adult female talkers were collected before and after two nights of restricted sleep and then statistical learning methods were applied to the audio to discriminate between the two occasions. In terms of originality the manuscript describes a new corpus unlike any existing corpus, and exploits a method for exploring which acoustic features were used by the classifier – a method I had not seen applied to this area before. In terms of innovation the method uses a variant of a previously developed modulation spectrum for feature extraction, and well developed methods for feature selection and pattern classification.

We would like to thank the Reviewer for their accurate description of the manuscript and their appreciation of its originality. We are also grateful for the specific and helpful comments provided (see below). We believe that they helped us enrich the manuscript and strike a better balance between the neuroscience and machine-learning implications of the work.

In terms of importance, the manuscript does not compare the new method with a baseline approach on the new corpus, so it is difficult to know whether the new methods are in improvement to existing approaches.

We acknowledge that the manuscript did not include present baseline results using machine-learning focused approaches. This shortcoming has now been fully addressed in a new Results section and new Figure 3, using the openSMILE framework that you kindly recommended, coupled with a pipeline from a state-of-the-art study which used a similar setting of participants reading texts (Krajewski et al., Speech Comm., 2016).

Details of the outcome are provided in response to your specific points 1, 2, and 3 below. In a nutshell, we find that the classification task on our database is not a trivial one. Accuracy with our original approach is on par or better than the openSMILE pipeline. We further emphasize, right from the first paragraph, that the goal of the manuscript is not to improve on the state-of-the art in terms of classification performance, but, rather, to provide a framework for *interpreting* current and future sleepiness detection algorithms.

In terms of insight, the manuscript speculates that there are two different mechanisms by which sleep restriction affects speech: a cognitive mechanism and a physiological mechanism – while this is almost certainly true, there is very little evidence in the experimental outcomes to be able to draw this conclusion.

We fully acknowledge that the link was speculative, which should have been made clearer. In the revised manuscript, following your comment and a similar one by Reviewer 1, this limitation is emphasized, starting from the fully reworked Abstract. Furthermore, alternative interpretations are provided in the Discussion.

In terms of rigour the method does have some significant weaknesses, in particular there is no cross-validation of individual speakers (unless I have misunderstood the method), so that the same

speaker can be present in both training set and test set; really the method needs to use a leave-one-speaker-out cross-validation to get an appropriate estimate of classification accuracy for an unknown speaker.

You are correct that the evaluation was done with a k-fold cross-validation technique, which included samples from all speakers in training and testing. We have now implemented the leave-one-subject-out (LOSO) cross-validation and report its results. Briefly, modest but still above change performance is observed with LOSO. Consistent with this new analysis, we now caution that generalization to an unknown speaker is not a likely use case of the method we present. We further point out that the outcome is consistent with the expected variability of the consequences of sleep deprivation across participants, motivating the individual approach taken in our study. Further details are provided in response to your point 1).

In terms of evidence for conclusions, the paper bases its understanding of the effect of sleep restriction on voice on a post-hoc method of correlating acoustic features with classifier outputs – however the classifier did not perform particularly well at detecting sleep restriction, and as mentioned, was not tested with appropriate cross-validation.

We hope that the methodological additions elicited by your comments have strengthened the evidence for the conclusions. In particular, while we clarify that classification is poor for unknown speakers, it is still on par with the state-of-the-art (see specific points 1 and 2). The individual classifier's performance remains excellent, strengthening our point that individual variability is key when considering vocal correlates of sleep deprivation.

Overall, the authors should think more carefully about what they want to show with this corpus with maybe a focus on what changes occur in the speech signal which correlate with sleep restriction and whether there is evidence for two separate processes.

We agree, and we believe that your comments have helped us further clarify the focus of the manuscript: interpreting sleep deprivation classifiers by relating their decisions to speech features, on a speaker-by-speaker basis. This is now stated right in the first paragraph of the Introduction. The Abstract has also been thoroughly reworked accordingly.

Here are some suggestions for improvements to the study:

We are truly grateful for these constructive suggestions, which we all implemented in full.

1. Introduce a leave-one-speaker-out cross validation to the method so as to establish a performance figure for speaker-independent sleep restriction detection.

We implemented a Leave-One-Subject-Out (LOSO) cross-validation procedure, so that the same speaker does not appear both in the training set and the test set. We agree that this is the gold standard to assess the generalizability of the classifier to unknown speakers. Results are now reported in the manuscript. The LOSO provided poor but still above-chance performance. Such an outcome is not a shortcoming of our classification pipeline: in response to your point 2), we also

implemented a state-of-the art pipeline based on openSMILE features. This provided similar LOSO results.

We also note that it has been argued that LOSO may not be appropriate for small datasets with large inter-subject variability (Varoquaux et al., 2018), a situation which applies to our own dataset. In fact, the study similar to ours that you kindly pointed out (Baykaner et al. 2015) also reports k-fold cross-validations. Thus, to present our results as transparently as possible and for comparison purposes, we present and discuss both the k-fold cross-validation results and LOSO cross-validation results.

Most importantly, the LOSO findings help us strengthen a point of the manuscript: large individual differences are expected in responses to sleep deprivation and its vocal correlates. The aim of our method is to interpret such differences at the individual level, and indeed, we found diverse patterns in individual classifiers. This surely explains the relatively poor LOSO performance, and cautions against the use of our classification pipeline to detect sleep deprivation in unknown speakers. This is now stated in the manuscript.

2. Consider introducing a state-of-the-art baseline method to establish the difficulty of the task. For example, OpenSMILE features and an SVM with leave-one-out cross-validation.

We have implemented exactly this suggestion, closely following Krajewski et al., Speech Comm., 2016. This is now described in the Method and Results sections of the manuscript. The pipeline used 4368 openSMILE features, followed by dimensionality reduction and an SVM. Overall, the openSMILE features front-end led to equivalent or worse classification performance compared to the STM front-end.

We hope that the inclusion of a state-of-the art baseline, which was clearly missing from the previous version of the manuscript, will reassure experts of the field that our conclusions are not dependent on the novel choices made for the classification pipeline.

3. Consider plotting some basic voice features as a function of sleep restriction, e.g. pitch height, pitch variation, speaking rate, breathiness, and creakiness.

We have followed this suggestion and added a new Figure 2D, comparing a selection of openSMILE features before and after sleep deprivation.

Specifically, we plot the openSMILE features we believe are the most directly related to the ones you suggested: pitch (f0_mean); pitch variation (f0_SD); creakiness (Jitter); and breathiness (logHNR). We omitted speaking rate as we could not find a direct correlate in the openSMILE features, and also as speaking rate is directly related to the Rate axis of Figure 2A, B, C. We could of course include further openSMILE features if you have other suggestions.

Briefly, the new Figure 2D visually shows that there are no obvious changes in the selected features before and after deprivation, complementing the STM analysis of Figure 2A,B,C. In particular, the direction of change is inconsistent across speakers for all features. Moreover, the features plotted

are part of the openSMILE baseline classifier, which displayed poor performance, confirming the difficulty of the classification task at the population level.

4. Look at how speech recordings vary within the day, not just before and after sleep restriction. Typically it is fairly easy to distinguish morning speech from evening speech, at least in a speaker dependent system. Differences within one day may be larger than the differences before and after sleep restriction.

We agree that differences within a day would be interesting to investigate, as was done in Baykaner et al. However, given our experimental design, we fear we do not have the necessary statistical power to examine within-day variations before sleep deprivation (only half the dataset) or the interaction between within-day variations and deprivation (second-order effect). We now mention this limitation in the Discussion and point to the Baykaner reference.

5. There is a directly relevant study by Baykaner et al [Baykaner KR, Huckvale M, Whiteley I, Andreeva S, Ryumin O, "Predicting Fatigue and Psychophysiological Test Performance from Speech for Safety-Critical Environments", Frontiers in Bioengineering and Biotechnology, 3, 2015] in which changes to speech over a period of sleep deprivation are tracked using support-vector-regression. This paper also uses modulation spectrum features. It would be useful to compare the findings of this study with results obtained on the new corpus. In particular looking at the problem as a regression problem rather than a classification problem would seem to open up more downstream applications. Indeed, the current title of the manuscript suggests that the current article "measures" sleep deprivation, whereas in fact it simply classifies speakers as being recorded before or after a period of restricted sleep.

Thank you for pointing out this highly relevant study and our apologies for the oversight in the previous version of the manuscript. We now discuss at length this study in the Discussion.

Briefly, we first note that they used a much more drastic deprivation procedure than ours (60 hours without sleep), so the present results extend their earlier findings. We then discuss the idea to use a regression approach instead of a classification one. Interestingly, one of their striking findings is that it seems very difficult to predict subjective reports from vocal features, likely because subjective reports do not correlate well with objective measures such as sleep onset or behavioral reaction times. So, the appropriate way to apply a regression model like the one used by Bayakaner et al. would be to try and predict objective and quantifiable correlates of sleep deprivation. Unfortunately, we do not have such quantifiable measures. As a result, we chose to describe this interesting approach in the Discussion.

Finally, we agree that the title may have been misleading. We changed it to: "Sleep deprivation detected from voice analysis".
