# Supplementary material for: Sleep deprivation detected by voice analysis
Source: PLoS Comput Biol. 2024 Feb 5;20(2):e1011849. doi: 10.1371/journal.pcbi.1011849 (PMC10890756; doi:10.1371/journal.pcbi.1011849)
Supplement: Attachment — Submitted filename: Sleep_voice_R2_rebuttal_submission.pdf [file pcbi.1011849.s006.pdf]

Rebuttal letter

PCOMPBIOL-D-23-00407

Reviewer #3: Andrey Anikin

Thoret et al. present a study that applies machine learning to the problem of detecting sleep deprivation from voice samples. First of all, I would like to stress that I really enjoyed reading the study. The methods are brilliant, and the acoustic and data analysis are of excellent quality. Using spectrotemporal modulation features and then reverse-correlating the classifiers are both original and promising techniques to add to the quiver of voice scientists. While I am not so familiar with literature on sleep deprivation, the theoretical grounding of the study and literature review seem very good as well.

First and foremost, we would like to warmly thank you for joining the review process at this late stage. And thank you also for this very positive appreciation of the methodological aspects and theoretical groundings of our work.

Unfortunately, I do see one major problem, namely the central claim that the presented models detect sleep deprivation per se – see the next comment. Perhaps the authors can refute this concern, and then all is well; but if not, then the results are in serious doubt.

The issue you raised was indeed critical. We believe we have different lines of evidence, including a completely novel analysis prompted by your comment, which reassured us (and we hope, will convince you as well) that the results do stand: sleep deprivation had an effect on the voice, above and beyond the natural variability of people reading the same text more than once.

MAJOR

line 511 "some of our participants responded to deprivation with less speech modulation, consistent with the classic "flattened voice" hypothesis (26, 27), whereas others unexpectedly responded with speech over-modulation and instead produced an "animated voice" after deprivation" A person reading the same text twice will presumably not have exactly the same intonation, speech rate, manner of articulation, etc. because they may also be generally more animated, tired, anxious and so on at the second reading (and not merely more sleep-deprived). Thus, some variation between the two readings is virtually guaranteed, and I don't see how it is possible to determine whether acoustic changes are caused by sleep deprivation or something else if (1) the direction of these changes varies across individuals and (2) each individual is recorded only once (before + after sleep deprivation). Specifically, the implicit assumption that each 15 s frame in a recording is an independent datapoint is problematic, and the per-individual classifiers in Fig. 3B can be interpreted as detectors of a particular recording rather than of sleep-deprived state.

This is clearly a point well taken. The question asked is a profound one, which should be considered for any experiments where individual variability is of interest: is the variability meaningful and related to the experimental manipulation, or is it just "noise"? In our case, the issue is compounded by the use of ML classifiers, which have the unfortunate knack to pick up on any source of variability to perform the task on which they are optimized.

Let us first provide some arguments to rule out "trivial" confounds such as noise stemming from the experimental conditions. From the sleep literature, we were fully expecting a large inter-individual variability, so we deliberately took every precaution to minimize noise in the recording procedure itself. Indeed, we would argue that our experiment is perhaps the most controlled yet in the voice-sleep literature: all participants were recorded with the same microphone before and after deprivation, in the same room, at the same location in the room. This minimizes confounds such as microphone frequency response or room reverberation, which could have been picked up by the classifiers. Furthermore, thanks to our interpretation method, we have an idea of which features were discovered by the classifiers. All features in the prosody domain, which came out of our data-guided analysis, cannot be ascribed to *e.g.* room acoustics or microphone filtering.

However, as you correctly point out, there is also the distinct possibility that people simply talk in different ways when reading the same text more than once, for reasons unrelated to sleep deprivation. Unlike the trivial confounds, this could perfectly well affect prosody and invalidate all of our points made about the effects of sleep deprivation on the voice.

As a first element of response for such a critical confound, we note that a significant correlation was observed between interpretation features related to prosody and subjective sleepiness (L 454-455 of the original manuscript). Sleepiness could also be predicted above chance from interpretation features (L 405-410). These two findings would be hard to explain if the experimental manipulation had no effect.

To prove that you detect specifically sleep deprivation, I think you would need either to demonstrate robust classification with LOSO (which you don't) or to record the same individual multiple times in normal/sleep deprived state (and thus prove that there are indeed subject-specific, but stable acoustic markers of sleep deprivation).

Thank you very much for suggesting such an elegant way out. To paraphrase, if we had more than one reading per participant, then we could compare the natural variability of repeated readings and the variability due to the experimental manipulation.

It turns out that we do have multiple recordings per participant: three reading sessions before deprivation and three reading sessions after deprivation, recorded at different times of the day (this was perhaps unclear, it is now clarified in the main text). All recordings were pooled together for the main results, but following your suggestion, we now ran an additional analysis.

We trained and cross-validated classifiers identical to those used in the main analyses (SVM+RBF), but this time to discriminate between all possible pairs of different reading sessions. We then averaged the average performance per subject for all sessions *within* the same state (discriminate two different sessions recorded both before or both after deprivation) or *across*

states (discriminate one session recorded before deprivation and one session recorded after deprivation). If our experimental manipulation had no effect, the accuracies "Within" and "Across" should be identical.

This was clearly not the case (see new Figure 4 of the revised manuscript, two-tailed comparison $t(21)=5.1$, $p=5e-5$). Note that this new analysis likely underestimates the effect of the experimental manipulation, because a lot of "Across" accuracies were near ceiling. Also, there are an interesting, non-random causes of variability within a state: the circadian rhythm and the homeostatic pressure for sleep that are both associated with fatigue and which are possibly picked up by the classifiers and could explain the relatively high performance already in the "Within" case.

We hope that you will find these new elements convincing. We do think that they bring a previously lacking piece of evidence to the story, for which we are grateful. We took the liberty to acknowledge your insight in the revised manuscript.

It's actually interesting that the population-level classifier (Fig. 3A) is fairly good at ~70%, but it could be because there aren't that many recordings and a vast number of predictors, so the same SVM model can learn to recognize multiple recordings from different variables (look at how different the individual classifiers are in Fig. 6A); if this interpretation is correct, the population-level accuracy should steadily decline as you increase the number of participants included in the model (as suggested by the better performance of per-individual models in Fig. 3B).

We agree with this interpretation, which is now mentioned when presenting the population level accuracies.

MINOR

Abstract "two distinct effects of sleep deprivation on the voice: a change in prosody and a change in timbre" In speech science it is more usual to talk about "voice quality" rather than "timbre". The term "prosody" may also need to be defined explicitly as different authors include different acoustic characteristics in it.

Done.

Because Methods are placed at the end of the paper, the key methods really need to be at least briefly explained in the Introduction and/or the Results. Personally, I was initially puzzled by the spectrotemporal modulation space, assuming you were talking about MPS until I checked the Methods section, because the present explanation (lines 142-145 and 211-221) is really brief. I suggest adding a more informative description along the lines of the paragraph "Spectro-Temporal Modulations of musical sounds" in Thoret 2021.

Sorry for the initial puzzlement. We indeed used our implementation of the STRF analysis of Chi et al. (2005), and not the MPS. This representation differs from the MPS mostly by its logarithmic frequency scale, but we believe that both representations would provide similar results in our case.

We have expanded the description of STMs/MPS when describing acoustic features in the main text, on L208-224.

Another key piece of information is what constitutes a datapoint - apparently, a 15 s frame, so each subject had between 98 and 194 datapoint. This is only mentioned on line 720, but without this information the reader may assume that there was in fact just one datapoint per recording, and then it's a mystery how the classifiers could be trained for each participant separately.

Thank you for pointing out this oversight. The description of the pipeline in the Methods has been clarified (L745). The definition of "frame" is also introduced early on in the main text (L172) and its usage has been harmonized throughout the manuscript – we previously used "samples", which we agree was confusing.

line 490 "The individual classifiers using the STM input features learnt to detect sleep deprivation with a high accuracy, matching human performance" This would be more convincing if coupled with a perceptual experiment performed on these recordings (or shorter parts thereof)

We added a reference that does contain such a perceptual experiment and clarified that we meant "human performance as previously measured in a similar experiment", and not for our own database.

line 731 "for each cross-validation procedure, we conducted a t-test against the threshold of 0.5 using the distributions of balanced accuracies." A more powerful test for both the 50-splits and LOSO would be a GLMM modeling binary classifications of each 15 s frame

We are not sure that we fully understand this last suggestion. In the manuscript, the t-tests were intended to formally compare the classifiers' performance to chance level. We had 50 different measures of classifier performance per test, because we ran 50 times the randomization procedure which split frames between the train and test sets. In a sense, all frames were used for each train-test run.

In contrast, as far as we understand, fitting a GLMM to predict the class of each frame would bypass the classifiers altogether and require using the auditory representation as an input to the GLMMs. To be fully transparent, this was actually our initial approach when we started the project. However, as we turned to ML classifiers and, crucially, to their interpretation rather than their accuracy, we gave up on the GLMMs.

In any case, we have reformulated l.731 to clarify the aim and implementation of the t-tests. If we have completely missed the idea behind your comment, please let us know and we'd be happy to implement another method to test the performance of the classifiers against chance.
